# A scalable tool for analyzing genomic variants of humans using knowledge graphs and graph machine learning

**DOI:** 10.3389/fdata.2024.1466391

**Published:** 2025-01-21

**Authors:** Shivika Prasanna, Ajay Kumar, Deepthi Rao, Eduardo J. Simoes, Praveen Rao

**Affiliations:** ^1^Department of Electrical Engineering and Computer Science, University of Missouri, Columbia, MO, United States; ^2^Department of Pathology and Anatomical Sciences, University of Missouri, Columbia, MO, United States; ^3^Department of Biomedical Informatics, Biostatistics and Medical Epidemiology, University of Missouri, Columbia, MO, United States

**Keywords:** knowledge graphs, human genomic variants, graph machine learning, scalability, inference

## Abstract

Advances in high-throughput genome sequencing have enabled large-scale genome sequencing in clinical practice and research studies. By analyzing genomic variants of humans, scientists can gain better understanding of the risk factors of complex diseases such as cancer and COVID-19. To model and analyze the rich genomic data, knowledge graphs (KGs) and graph machine learning (GML) can be regarded as enabling technologies. In this article, we present a scalable tool called VariantKG for analyzing genomic variants of humans modeled using KGs and GML. Specifically, we used publicly available genome sequencing data from patients with COVID-19. VariantKG extracts variant-level genetic information output by a variant calling pipeline, annotates the variant data with additional metadata, and converts the annotated variant information into a KG represented using the Resource Description Framework (RDF). The resulting KG is further enhanced with patient metadata and stored in a scalable graph database that enables efficient RDF indexing and query processing. VariantKG employs the Deep Graph Library (DGL) to perform GML tasks such as node classification. A user can extract a subset of the KG and perform inference tasks using DGL. The user can monitor the training and testing performance and hardware utilization. We tested VariantKG for KG construction by using 1,508 genome sequences, leading to 4 billion RDF statements. We evaluated GML tasks using VariantKG by selecting a subset of 500 sequences from the KG and performing node classification using well-known GML techniques such as GraphSAGE, Graph Convolutional Network (GCN) and Graph Transformer. VariantKG has intuitive user interfaces and features enabling a low barrier to entry for KG construction, model inference, and model interpretation on genomic variants of humans.

## 1 Introduction

Started in 1990, the Human Genome Project (Olson, 1993) undertook an ambitious effort to sequence the entire human genome to produce an official gene map. Over the years, the gene map has offered crucial insights into the human blueprint, accelerating the study of human biology and advancements in medicine. In recent years, genomics has been regarded as a Big Data science (Stephens et al., 2015). It is projected that between 100 million and 2 billion humans could be sequenced by 2025 producing between 2 and 40 exabytes of genome data. Whole-genome sequencing (WGS) has become economically feasible in large-scale studies and clinical practice. Improved understanding of the human genome has become the quintessential scientific process to treating life-threatening diseases and improving health outcomes. Variant calling is a fundamental task that is performed to identify variants in an individual's genome compared to a reference human genome. This task can enable better understanding of an individual's risk to diseases and eventually lead to new innovations in precision medicine and drug discovery.

Variants are genetic differences between healthy and diseased tissues or between individuals of a population. The process of analyzing these genetic differences or variations in deoxyribonuclei acid (DNA) sequences and categorizing their functional significance is called variant analysis. Ribonucleic acid (RNA) sequencing is similar to DNA sequencing but differs in its extraction technique. RNA is extracted from a sample and then reverse transcribed to produce what is known as copy or complementary DNA called cDNA. This cDNA is then fragmented and run through a next-gen sequencing system. Examining DNA provides a static picture of what a cell or an organism might do, but examining RNA tells us precisely what the cell or organism is doing. Another advantage of RNA sequencing is that molecular features sometimes can only be observed at the RNA level.

Variant calling pipeline is the process of identifying variants from sequence data. To measure the deleteriousness of a variant, the combined annotation dependent depletion (CADD) (Rentzsch et al., 2019, 2021) scores can be used. CADD evaluates or ranks the deleteriousness of a single nucleotide, insertion, and deletion variants in the human genome.

Knowledge graphs such as Yet Another Great Ontology (YAGO) (Mahdisoltani et al., 2013), Wikidata (Vrandečić and Krötzsch, 2014), DBPedia (Lehmann et al., 2015), and Schema.org (Guha et al., 2016) are crucial for structuring and linking vast amounts of diverse data, to enable efficient information retrieval, enhance data interoperability, and provide a foundation for advanced applications in domains not limited to semantic search, natural language processing (NLP), and data integration. One such example is the work of Dong (2018), which focuses on constructing a comprehensive KG called ProductKG from Amazon's large-scale and diverse product catalog. ProductKG captures product attributes, categories, and relationships using graph mining and embedding techniques. This structured representation aims to improve understanding and retrieval of product information, thereby enhancing user experience and supporting various artificial intelligence (AI) applications within Amazon's ecosystem. Another such example is FoodKG, introduced by Gharibi et al. (2020) that demonstrates the importance of knowledge graphs in the food domain through their tool, FoodKG, by integrating diverse datasets, using NLP to extract meaningful entities and state-of-the-art model to enhance and enrich graphs based on food, energy, and water (FEW) used by the tool. Their contribution highlights the significant role of KGs in managing and utilizing large-scale, heterogeneous data.

Representing genomic data as KGs allows vast and diverse information from various sources to be integrated. These specialized graph structures, which model entities as nodes and relationships as edges, provide an ideal framework for integrating and organizing diverse biological information from multiple sources. Furthermore, it allows for efficient querying and indexing and supports inference and new knowledge discovery.

The key contributions of this work are as follows:

- We developed VariantKG, a scalable tool that represents human genome variants as a KG in RDF. VariantKG can consume a large number of variant call format (VCF) files produced by a variant calling pipeline and annotate them using SnpEff (2024) to generate additional information about the raw variants. The annotated files were converted to the RDF format using Sparqling-genomics (Di Bartolomeo et al., 2018).
- VariantKG employs a new ontology for the genomic variants to precisely represent the variant-level information. It leverages Wikidata (Vrandečić and Krötzsch, 2014) concepts that are useful for representing genomic data. It also extracts patient metadata (e.g., age, sex, disease stage) from the European Nucleotide Archive (ENA) browser. Additional RDF statements are then inserted into the KG based on these resources making the underlying KG unique and valuable.
- A key feature of VariantKG is the synergistic integration of GML for conducting inference tasks on the KG. It employs DGL for training and inference on the KG. A user can select a subset of the KG and prepare the data for GML. They can monitor the training and testing performance such as accuracy, loss, and hardware utilization. Currently, VariantKG supports node classification tasks on the KG using well-known techniques such as GraphSAGE (Hamilton et al., 2017), GCN (Zhang et al., 2019), and Graph Transformer (DGL Team, 2024). It also supports the model interpretability where it extracts the top performing features used in model training and also, provides the subgraphs and node features crucial for predicting the target node.
- VariantKG was evaluated by constructing a KG with 4 billion RDF statements from 1,508 VCF files. Each VCF file required between 1 and 3 min to process. We evaluated VariantKG for GML tasks by selecting a subset of 500 VCFs and their additional metadata from the KG for node classification. In short, VariantKG enables low barrier to entry for users interested in GML on human genomic variants.

The remainder of the article is organized as follows: Section 2 presents related work on KGs for biomedical data and GML; Section 3 presents the overall design and implementation of VariantKG including data processing pipeline, KG construction using a new ontology and other relevant metadata, and GML for node classification tasks. Section 4 presents a case study on how the tool was used to model the genomic variants of COVID-19 patients and perform inference tasks using GML (i.e., GraphSAGE, GCN and Graph Transformer) along with model interpretability. Finally, we conclude in Section 5.

## 2 Related work

### 2.1 Prior studies on KGs

KGs are used to integrate and analyze diverse genomic data, providing a comprehensive and contextual representation of genomic information. For instance, Feng et al. (2024) extended the development of GenomicKB by creating a graph database that integrates the human genome, epigenome, transcriptome, and 4D nucleosome data. This extensive database, annotated from over 30 consortia and portals, includes 347 million entities, 1.36 billion relations, and 3.9 billion properties, covering comprehensive data on pancreases and diabetes' genome-wide association study (GWAS), disease ontology, and expression quantitative trait loci (eQTL) data. Another notable work (Feng et al., 2023) presented a knowledge graph, GenomicKB, that consolidates various sources of genomic information, including data from genomic databases, experimental studies, literature, and public repositories, into a single, unified framework. This integration facilitates efficient data analysis and knowledge discovery through a user-friendly web portal.

KG exploration and visualization have become crucial in biomedical research, with several frameworks addressing various aspects of the challenge like KGEV (Peng et al., 2022) offering flexible framework that combines elements of knowledge graph construction, interactive visualization, and rapid access to supporting literature. It is a five-stage pipeline for graph construction and visualization with best practices in the field. KGs have been well integrated with biomedical data specifically for precision medicine. AIMedGraph (Quan et al., 2023) builds upon such efforts that developed an evidence-based knowledge graph that uniquely focuses on variant-drug relations, addressing a critical gap in precision medicine knowledge representation.

KGs have been instrumental in understanding the COVID-19 pandemic and its treatment. For example, Sakor et al. (2023) developed a framework that integrates diverse COVID-19 drug resources to discover drug-drug interactions among COVID-19 treatments, utilizing RDF mapping language and NLP to extract relevant entities and relationships. Similarly, Reese et al. (2021) proposed KG-COVID-19, a knowledge graph framework that integrates heterogeneous data on SARS-CoV-2 and related viruses, supporting downstream tasks such as machine learning, hypothesis-based querying, and user interface exploration. Chen et al. (2021) used RDF to integrate COVID-19 data extracted from iTextMine, PubTator, and SemRep biological databases into a standardized KG. This COVID-19 KG supports federated queries on the Semantic Web and is accessible through browsing and searching web interfaces, with a RESTful API for programmatic access and RDF file downloads.

These diverse efforts highlight the significant potential of integrating KGs with genomic data and deep learning, facilitating comprehensive data integration, efficient analysis, and innovative solutions to complex problems in genomics and beyond.

### 2.2 Prior studies on GML

Recent advancements in predicting the emergence of COVID-19 variants have led to innovative approaches using graph-based machine learning techniques. Aawar et al. (2024) introduced a novel variant-dynamics-informed graph neural network (GNN) for global prediction of the COVID-19 variant spread. Their method incorporates a derived model of variant prevalence dynamics between countries, addressing the limitations of previous statistical approaches. The authors developed a comprehensive benchmarking tool covering 87 countries and 36 variants, enabling rigorous evaluation of prediction models. Their dynamics-informed GNN outperformed baseline models, including physics-informed neural networks (PINNs), in retrospectively predicting variant emergence and delay.

Song et al. (2023) explored the application of GNNs to predict COVID-19 infection patterns. The authors developed an innovative approach using GNNs to predict the influence of infected individuals on future infections. Their study utilized a comprehensive dataset containing interaction information between the confirmed cases, including contact order, times, and movement routes. The authors compared two GNN variants, GCN and graph attention networks (GAT), against traditional machine learning models. Their results demonstrated that graph-based models significantly outperformed conventional approaches, with improvements in area under the curve for second, third, and fourth order spreading predictions by 0.200, 0.269, and 0.190, respectively. This research underscores the importance of incorporating relational data between infected individuals in epidemiological modeling and suggests that graph-based approaches can substantially enhance the effectiveness of automatic epidemiological surveys.

Harnoune et al. (2021) proposed constructing KGs from clinical data using the Bidirectional Encoder Representations From Transformers (BERT) model. This approach focused on creating biomedical knowledge graphs by leveraging BERT's contextual understanding capabilities to process biomedical text data, including clinical records and scientific literature, extracting meaningful and contextually rich information. Domingo-Fernández et al. (2021) developed a multi-modal cause-and-effect COVID-19 knowledge model using biological expression language (BEL) as a triple (i.e., source node-relation-target node) with metadata about nodes. This model utilized GraphML, NDEx, and SIF representations for network visualization and was made accessible through a web platform to enhance its visibility and utility.

Deep learning has significantly influenced a wide range of domains, with genomic studies being particularly impacted. For instance, Liu et al. (2020) introduced DeepCDR, a method using deep learning to predict cancer cells' response to different drugs, facilitating effective cancer treatment. Another innovative approach was proposed by Lanchantin and Qi (2019), who developed ChromeGCN for predicting epigenetic states using sequences and 3D genome data. ChromeGCN leverages GCNs to predict the epigenetic states of genomic regions, representing genomes as graphs where nodes are genomic regions and edges represent relationships between them. This method's predictive power enables the identification of functional genomic elements and regulatory regions, providing insights into gene regulation and cellular function.

Al-Obeidat et al. (2020) focused on extracting and utilizing knowledge from COVID-19-related news articles, providing a platform for researchers, data analysts, and data scientists to investigate and recommend strategies to address global challenges.

Finally, Sun et al. (2018) proposed kernelized generalized Bayesian rule mining with support vector machines (KGBSVM), a method to analyze high-dimensional genome data, aiming to improve classification accuracy on general tasks, whether binary or multi-class. This method enhances the accuracy and efficiency of genomic data classification, contributing to better data analysis and interpretation in the field.

A summary of closely related work on KGs and GML is shown in Table 1.

**Table 1:** Summary of related work on KGs and GML in genomics and biomedical research.

**Reference**	**Problem addressed**	**Methods used**	**Key contributions**
Feng et al. (2024)	Genomic data integration	Graph database integrating genome, epigenome, transcriptome, and 4D nucleosome data	Created a database with 347M entities, 1.36B relations, and 3.9B properties
Feng et al. (2023)	Consolidation of genomic information from several sources	KG construction integrating data from genomic databases, experimental studies, literature, and public repositories	Facilitated data analysis and knowledge discovery through a unified framework and web portal
Peng et al. (2022)	KG exploration and visualization in biomedical research	KGEV framework combining KG construction, interactive visualization, and rapid access to supporting literature	Offered a flexible five-stage pipeline for graph construction and visualization with best practices
Aawar et al. (2024)	Predicting COVID-19 variant emergence	Variant-dynamics-informed GNN	Developed a comprehensive benchmarking tool covering 87 countries and 36 variants; outperformed baseline models in predicting variant emergence
Song et al. (2023)	Predicting COVID-19 infection patterns	GCNs and graph attention networks (GAT)	Demonstrated significant improvements in predicting infection spread compared to traditional machine learning models

## 3 Design and implementation of VariantKG

In this section, we present the overall design and implementation of VariantKG. First, we explain the data processing pipeline, followed by the construction of KGs using a new ontology and other relevant metadata. Then, we elaborate on the use of GML for node classification tasks.

### 3.1 Data preprocessing pipeline

**Figure 1:**
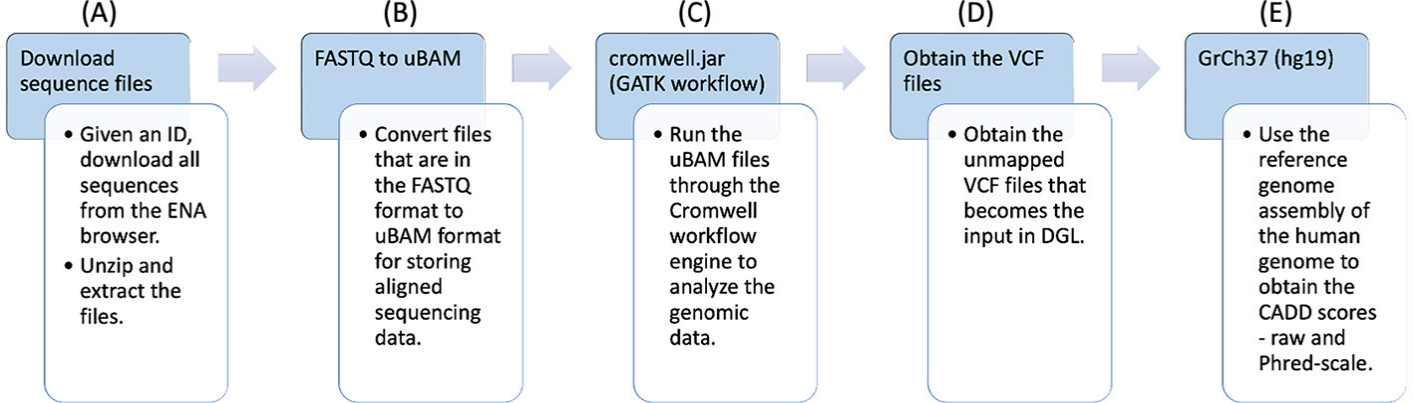
Workflow for preparing the genomic variants dataset from raw genome sequences.

COVID-19 RNA sequences IDs were first collected from the ENA. The workflow is shown in Figure 1.

- FASTQ files (part A): These IDs were utilized to download the RNA sequences, that were in FASTQ (Li et al., 2008; Li and Durbin, 2009) format. The FASTQ file format consists of a series of records, each of which contains four lines of text: the first line starting with “@” contains a sequence identifier, the second line contains the actual nucleotide sequence, the third line starts with ‘+' and may optionally contain additional information about the sequence, and the fourth line contains quality scores encoded as ASCII 10 characters. The quality scores indicate the confidence in the accuracy of each base call and are typically represented as Phred scores.
- uBAM files (part B): The FASTQ files were then converted to unmapped binary alignment map (uBAM) files for storing aligned sequencing data. A uBAM file contains unmapped reads. These reads can be used for downstream analysis, such as de novo assembly, quality control, and identification of novel sequences.
- GATK workflow (part C): The uBAM files were passed through the genomic analysis toolkit (GATK) workflow (McKenna et al., 2010) that converts the files into variant calling format (VCF) (Danecek et al., 2011) files. It is a comprehensive toolkit developed by the Broad Institute that includes various tools and algorithms for processing genomic data, such as read mapping, local realignment, base quality score recalibration, variant calling, and variant filtering.
- Pipeline output (part D, part E): The unannotated VCF files that were obtained as the result of the workflow have been shown in part D. For each VCF file, there is also a corresponding CADD Scores file that was obtained using genome reference (GrCh37) through the workflow, as shown in part E.

We have implemented several optimizations to enhance the efficiency and scalability of our data processing pipeline. By leveraging the ThreadPoolExecutor, we have parallelized the SNP annotation process, significantly improving performance for complex and large datasets. Our pipeline has been rigorously tested with varying numbers of VCF files, ranging from 50 to 1000, with file sizes between 1 and 17MB. These tests have demonstrated robust performance across different scales. For more details, refer to Section 4. The parallelization of workflow steps allows for more efficient utilization of computational resources, enabling the processing of larger and more intricate datasets. While our current tests have shown excellent performance with up to 1000 VCF files, the architecture is designed to scale further, limited only by the available computational resources. These enhancements ensure that our pipeline can effectively handle the increasing volume and complexity of genomic data, providing a solid foundation for future expansions in data processing capabilities.

### 3.2 Data annotations

Once the workflow was executed, two main files were generated for each RNA sequence ID: a VCF file and a CADD Scores file.

**Figure 2:**
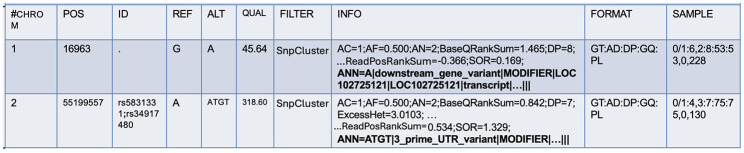
Additional annotations produced by the SnpEff tool.

For further annotations, SnpEff (Cingolani et al., 2012), a command-line, variant annotation, and effect prediction tool was utilized. This tool annotates and predicts the effects of genetic variants. SnpEff classifies variants as single nucleotide polymorphisms (SNPs), insertions, deletions, multiple-nucleotide polymorphisms (MNPs), or an InDel. SnpEff takes the predicted variants (SNPs, insertions, deletion, and MNPs) as input and produces a file with annotations of the variants and the effects they produce on known genes. While the original VCF file contains the INFO field, SnpEff adds additional annotations to this field to describe each variant further. In the process, it also updates the header fields. This field is tagged by “annotations (ANN)”, which is pipe symbol separated and provides a summary of the predicted effects of a genetic variant on each affected transcript. Figure 2 shows the ANN field highlighted in bold.

A variant may have one or more annotations, and multiple annotations are comma-separated. There are several fields within the ANN tag, mainly:

- Allele (ALT): information on alternate alleles that are predicted to cause a functional impact on a gene or protein.
- Annotation (effect): type of effect caused by the variant on the transcript.
- Putative impact: qualitative measure of the impact of the variant on the transcript.
- Gene name: name of the affected gene.
- Gene ID: unique identifier of the affected gene.

### 3.3 Ontology

A KG is represented using an ontology, which can be represented using a formal language such as RDF, OWL (web ontology language), or another domain-specific language. The ontology in this study has been represented using RDF. Each node-edge-node is represented as a triple by RDF. In a triple, the subject defines the first node, and the object defines the second node. The predicate defines the edge or relation joining the two nodes. A triple always ends with a period (“.”).

**Figure 3:**
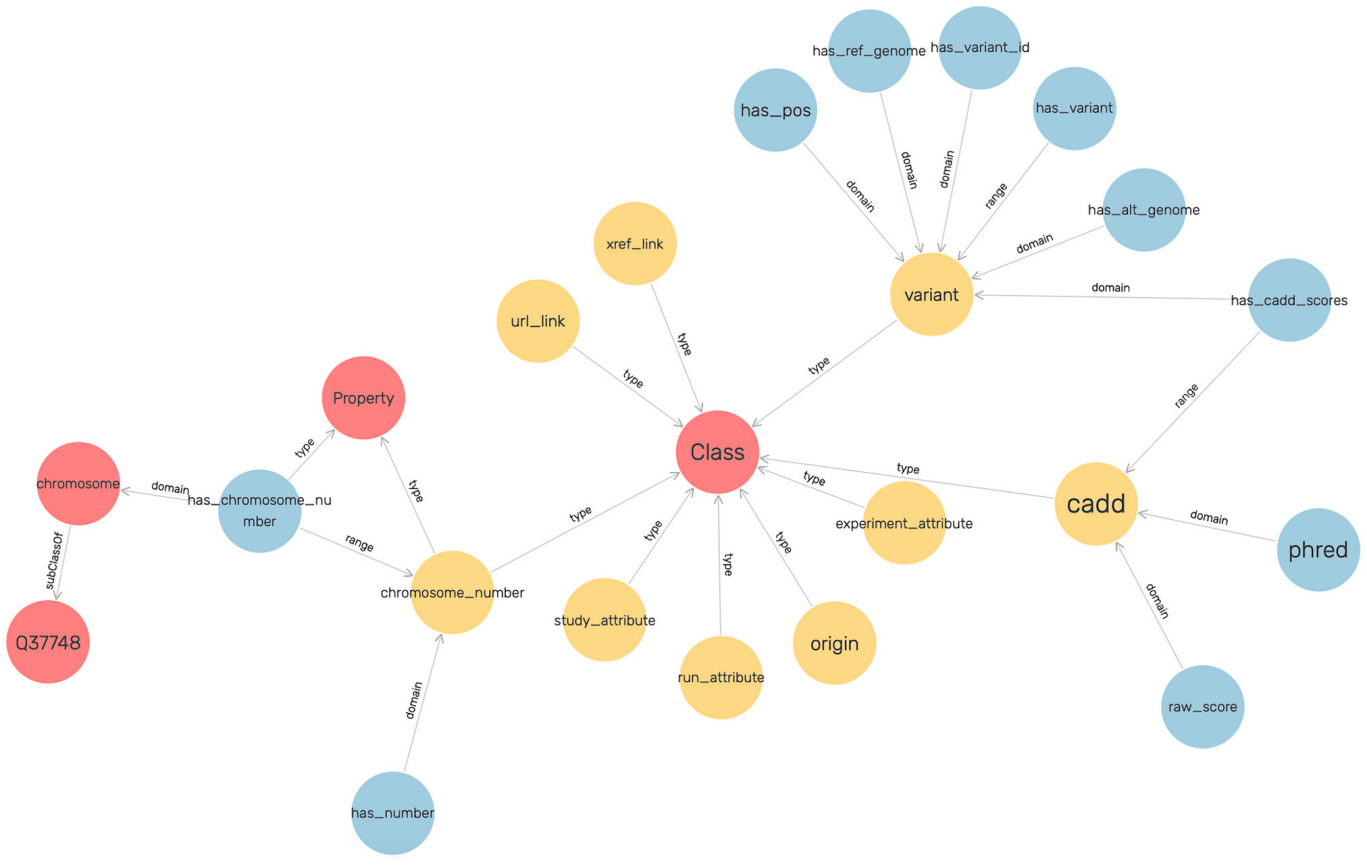
The ontology for modeling the genomic variants as a KG.

An ontology mainly consists of classes, properties, and relationships. We have explicitly defined an ontology to provide structure to genomic data in the KGs. This ontology can be easily expandable and flexible with additional variant information. In Figure 3, the sub-classes are depicted by red nodes, the classes are depicted by yellow nodes, and the relations by blue nodes. The description of the classes has been given in Table 2, and the description of the properties has been given in Table 3.

**Table 2:** Description of the classes in the ontology.

**Class**	**Definition**
Chromosome number	Identifier of the chromosome; values can be “1”, “2”, …, “22”, “X”, “Y”, “MT”
Origin	Unique identifier of the variant annotated by SPARQLing Genomics tool
Variant	Encapsulates the different types of genomic alterations that can occur
CADD	Encapsulates the different types of scores that can occur
xref_link	Type of annotation that provides a link between different resources or databases
url_link	Access link to experiment label
study_attribute	Metadata that describes the experimental design, data processing, and other aspects of a sequencing study
run_attribute	Metadata that describes the sequencing run
experiment_attribute	Metadata that describes the overall experimental design and goal of the experiment

**Table 3:** Description of the properties in the ontology.

**Property**	**Definition**	**Domain**	**Range**
has_pos	Variant position	Variant	Integer
has_ref_genome	Reference genome at that position	Variant	String
has_alt_genome	Alternate genome at that position	Variant	String
has_variant_id	Unique identifier of the variant	Variant	String
has_variant	Unique name given to the variant	Variant	String
has_cadd_scores	Variant has associated CADD Scores	Variant	CADD
has_chromosome_number	Chromosome has a chromosome number	Chromosome	String
phred	Phred-scaled score	CADD	Long
raw_score	Raw CADD Score	CADD	Long

In the defined ontology, chromosome and variant are both domain classes, and a chromosome has an associated chromosome number to connect all similar chromosomes as an extension, and a variant has an associated variant ID. A variant has a reference and alternate genome.

The ontology also explicitly defines CADD as a class where a variant has CADD Scores represented by both raw score and Phred-scale score, as properties of the CADD class. The ontology description is provided in Table 4.

**Table 4:** Domain, properties, and ranges for the ontology.

**Entity**	**RDF: property**	**Domain**	**Range**
Chromosome	Type	N/A	Wiki:Q37748
	SubClassOf	N/A	Wiki:Q37748
has_chromosome_number	Type	N/A	Property
	Domain	Chromosome	N/A
	Range	chromosome_number	N/A
chromosome_number	Type	N/A	Class
has_number	Type	N/A	Property
	Domain	chromosome_number	N/A
	Range	xsd:int	N/A
Variant	Type	N/A	Class
has_variant	Type	N/A	Property
	Domain	Chromosome	Variant
	Range	Variant	N/A
has_pos	Type	N/A	Property
	Domain	Variant	xsd:string
	Range	xsd:int	N/A
has_ref_genome	Type	N/A	Property
	Domain	Variant	xsd:string
	Range	xsd:string	N/A
has_alt_genome	Type	N/A	Property
	Domain	Variant	xsd:string
	Range	xsd:string	N/A
CADD	Type	N/A	Class
has_cadd_score	Type	N/A	Property
	Domain	Variant	CADD
	Range	CADD	N/A
raw_score	Type	N/A	Property
	Domain	CADD	xsd:long
	Range	xsd:long	N/A
phred	Type	N/A	Property
	Domain	CADD	xsd:long
	Range	xsd:long	N/A

### 3.4 Metadata

The metadata was downloaded from the Sequence Read Archive (SRA)^1^ web tool of NCBI. The metadata for each patient are also stored. We leveraged the SRA web tool over Entrez to extract a broader range of metadata information such as disease stage, age, sex, and tissue. The obtained metadata was then converted to N-Quad (NQ) triples to facilitate the use of RDF named graphs to link this information to the variant information obtained. An example of the metadata obtained for the accession ID SRR12570493 is as follows:


 
  SRR12570493,65 (Age),,RNA-Seq,119,1473875214,PRJNA661032,SAMN15967295,582422941,,GEO,Severe COVID,Alive,public,‘‘fastq,sra,run.zq'',‘‘gs,ncbi,s3'',‘‘s3.us-east-1,gs.US,ncbi.public'',SRX9058173,NextSeq 500,GSM4762164,,PAIRED,cDNA,Homo sapiens,TRANSCRIPTOMIC,ILLUMINA,2021-01-27T00:00:00Z,,2020-09-02T12:06:00Z,1,GSM4762164,male,6,Patient 14 blood,SRP279746,Patient 14,Blood,,,,,,,,,,,,
 


The NQ triple for the age attribute for the same accession ID run is as follows:


 
      < https://www.ncbi.nlm.nih.gov/sra/?term=SRR12570589>  < https://www.wikidata.org/wiki/Q11904283> ‘‘61.0''^^ < http://www.w3.org/2001/XMLSchema#float>  < sg://SRR12570589> .
 


### 3.5 Conversion of VCF files to KGs

To transform the VCF data into RDF, SPARQLing Genomics (Di Bartolomeo et al., 2018) was utilized. SPARQLing Genomics is an open-source platform for querying and analyzing genomic data using the Semantic Web and Linked Data technologies. The platform has been built to support SPARQL queries and various SPARQL query features, including sub-queries, filters, and aggregates along with an easy-to-use interface. SPARQLing Genomics provides several in-built, ready-to-use tools, one of which is vcf2rdf that converts VCF data into RDF triples.

The triples generated by the tool consist of uniquely identifiable names with symbolic and literal values like numbers or text. The subject and object are typically represented as uniform resource identifiers (URIs). The following is an example of a variant from the VCF file.


 
  #CHROM POS ID REF ALT QUAL FILTER INFO sample
     1  16963  .   G   A  45.64 SnpCluster AC=1;AF=0.500;AN=2;BaseQRankSum=1.465;DP-8;ExcessHet=3.0103;FS=0.000;MLEAC=1;MLEAF=0.500;MQ=60.00;MQRankSum=0.000;QD=5.70;ReadPosRankSum=-0.366;SOR=0.169GT:AD:D:GQ:PL0/1:6,2:8:53:53,0,228
 


The following shows the final triple output by SPARQLing Genomics for a variant position in the example shown above.


 
       < origin://4a37140cdc877d90ffe258a8151f27e@0>  < http://biohackathon.org/resource/faldo#position> ‘‘16963''^^ < http://www.w3.org/2001/XMLSchema#integer> .
 


As seen in the above example, the variant position is described with feature annotation location description ontology (FALDO) (Bolleman et al., 2016). For other features not defined by FALDO, the URI is customized by the tool.

Each VCF file eventually corresponds to one large KG that was originally stored in the N3 format, which is one of several formats supported by RDF and can be considered a shorthand non-XML serialization of RDF models. However, to accommodate the accession ID that would map to a de-identified patient, the N3 serialization was converted to NQ format with the accession ID as the named graph. An example of a triple from an N3 file is given below. The ontology was extended to accommodate the new relations generated by the tool.


 
       < origin://4a37140cdc877d90ffe2b58a8151f27e@0>  < sg://0.99.11/vcf2rdf/variant/REF>  < sg://0.99.11/vcf2rdf/sequence/G>.
 


The triple was then converted to the NQ format, which yielded the following:


 
       < origin://4a37140cdc877d90ffe2b58a8151f27e@0>    < sg://0.99.11/vcf2rdf/variant/REF>  < sg://0.99.11/vcf2rdf/sequence/G>  < sg://SRR13112995>.
 


### 3.6 Conversion of CADD Score files to KGs

The SnpEff and vcf2rdf tools were useful for converting VCF files to triples. However, the CADD Scores obtained through the pipeline were in tab-separated (TSV) format. To enrich the KGs, the CADD Scores had to be translated to RDF triples as well. Therefore, the ontology for CADD Scores was explicitly defined.

**Figure 4:**
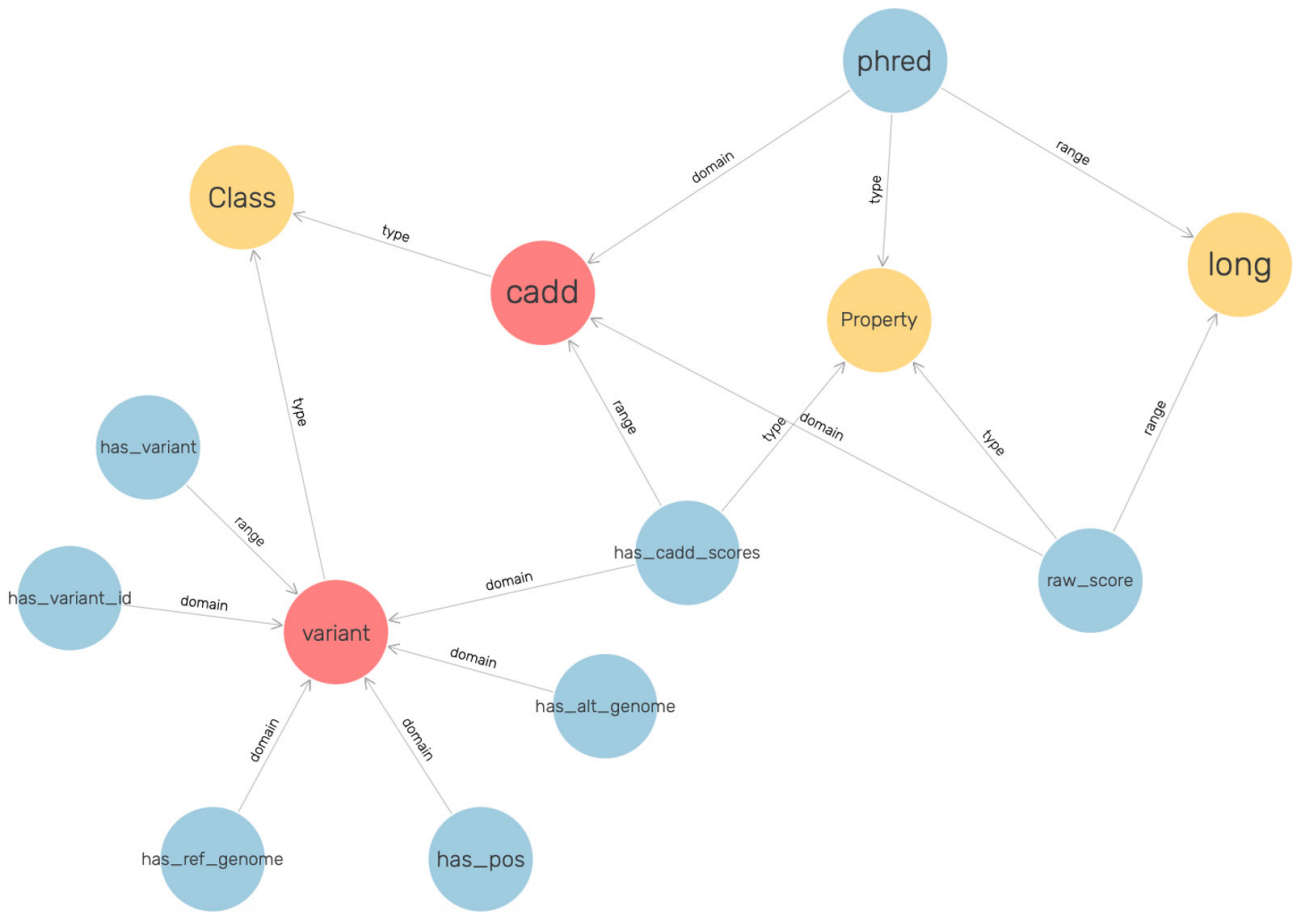
The ontology for modeling the CADD scores.

To visualize the graph, GraphDB was utilized, and the ontology that was defined for CADD Scores is shown in Figure 4.

These scores have been represented with respect to the fields described in the VCF files, such as the chromosome, position, reference genome, and alternate genome. The raw scores and Phred-scale scores were obtained from the original TSV files.

The following is an example of a record in a TSV file for which the raw and Phred scores map to Chromosome 1 with position 16963, reference base(s) “G” and alternate base(s) “A” in the VCF file:

**Table:** 

#Chrom	Pos	Ref	Alt	RawScore	PHRED
1	16963	G	A	0.900784	12.72

Each data record, like the above example, was converted to a Turtle triple (TTL), another format supported by RDF. A TTL format writes a graph in a compact textual form. A triple has three parts - subject, predicate, and object followed by the period. An example of the above data record converted to a TTL triple is given below:


 
      < http://sg.org/SRR13112995/1/variant1> a ns1:variant ;
          ns1:has_alt_genome ‘‘A'' ;
          ns1:has_cadd_scores  < http://sg.org/SRR13112995/1/variant1/cadd> ;
          ns1:has_pos 16963 ;
          ns1:has_ref_genome ‘‘G'' .
 


### 3.7 Graph storage and database

Each VCF file is represented as a single KG. So to unify several KGs into one single large graph, Blazegraph DB (2024) has been leveraged.

BlazeGraph is a high-performance, horizontally scalable, and open-source graph database that can be used to store and manage large-scale graph data. It has been designed to provide efficient graph querying and supports the RDF data model which allows it to store and process both structured and semi-structured data. BlazeGraph uses a distributed architecture that can be easily integrated with other big data tools, such as Hadoop^2^ and Spark,^3^ to perform complex analytics on large-scale graph data.

BlazeGraph has been leveraged to efficiently query the KGs to generate the dataset for GML node classification tasks. Other tools such as RIQ (Katib et al., 2017, 2016; Slavov et al., 2015) can be used to index and query RDF-named graphs.

The total number of triples in the KG, after aggregating only 511 VCF files on a single machine, was 3.1 billion. Hence, an efficient system such as BlazeGraph is necessary.

### 3.8 Deep graph library

To enable GML tasks, Deep Graph Library (DGL) (Wang et al., 2019), an open-source library supporting graph-based deep learning was utilized. DGL provides a set of high-level APIs for building scalable and efficient graph neural network models. With DGL, we can create, manipulate, and learn from large-scale graphs with billions of nodes and edges.

There are three main tasks supported by DGL:

- Node classification: Predict the class or label of a node in a graph based on its features.
- Link prediction: Predict if there is a relation or an edge between two nodes.
- Graph classification: Classify an entire graph into one or more classes or categories.

DGL represents a graph as a DGLGraph object, which is a framework-specific graph object. It takes two input parameters, namely, the number of nodes, and a list of source and destination nodes, where nodes and edges must have consecutive IDs starting from 0. Since DGL only accepts numeric input, all strings, such as URI, were mapped to integers. In this work, node classification was used to classify variants into CADD Score categories based on their features.

### 3.9 Node classification task

For this task, GCN (Zhang et al., 2019), GraphSAGE (Hamilton et al., 2017), and Graph Transformer DGL Team (2024) have been used. Each node is associated with a feature vector.

GCNs use node embeddings and adjacency matrices to compute new embeddings while training. Similar to convolutional neural networks (CNNs), the model weights and biases are first initialized to 1, and then a section of the graph is passed through the model. A non-linear activation function is used to compute the predicted node embeddings for each node. Cross entropy loss is calculated to quantify the difference between the predictions and the ground truth. For this task, loss gradients are then computed to update the model using the ADAM optimizer (Kingma and Ba, 2014). These steps are repeated until convergence.

GraphSAGE uses SAGEConv layers where for every iteration, the output of the model involves finding new node representation for every node in the graph. Mean is used as the aggregation function along with ReLU activation function. The ADAM optimizer was used for this model as well. One of the most noted properties of GraphSAGE is its ability to aggregate neighbor node embeddings for a given target node. This property was observed through the experiments conducted. GraphSAGE also generalizes better to unseen nodes because of its ability to perform inductive learning on graphs.

**Figure 5:**
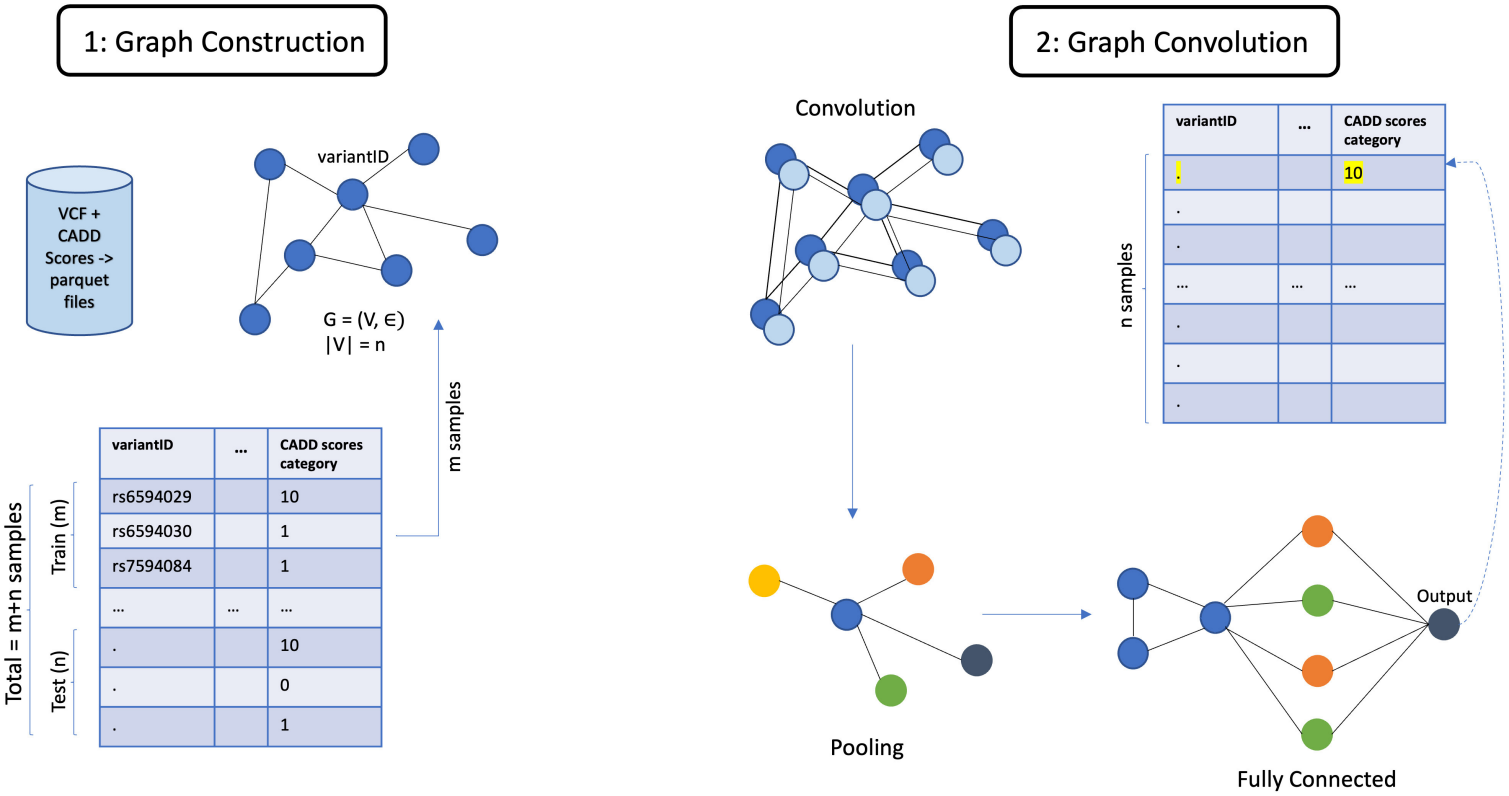
Architecture of GCN.

**Figure 6:**
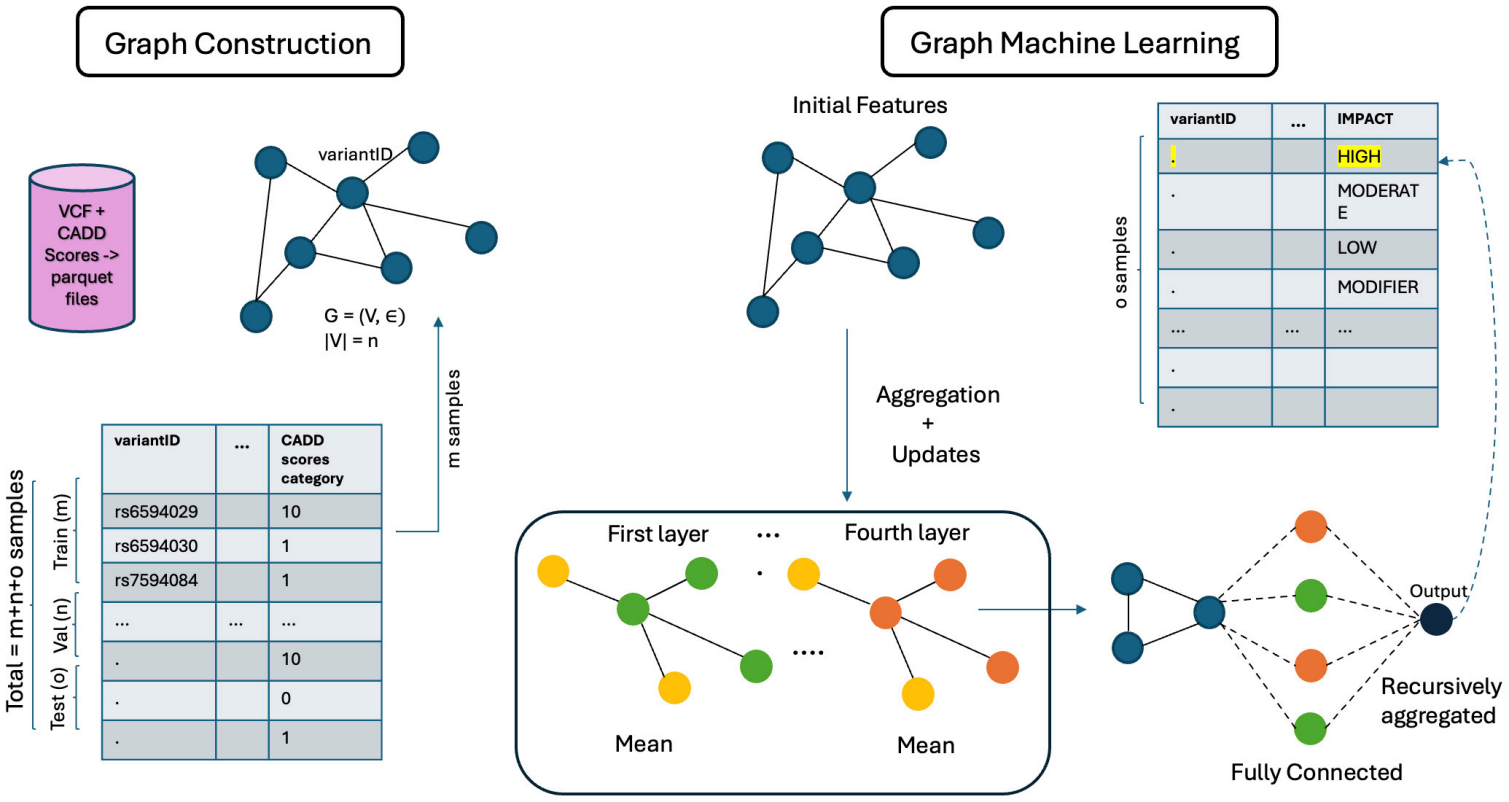
Architecture of GML.

The architecture of GCN is shown in Figure 5. The general architecture of GML, shown in Figure 6, differs in the property of message passing between the nodes. This was crucial as the nodes in the input graph relied on several pieces of information from their neighboring nodes. The ability to capture long-range dependencies in graphs while maintaining computational efficiency makes Graph Transformer a promising approach.

### 3.10 Model interpretability for GNN

GNN models are regarded as interpretable models because they facilitate learning about entities, relations, and rules for composing them. Entities are discrete and represent high-level concepts or knowledge items, and it is easier to find the explicit reasoning path or subgraph that contributes to the prediction. In VariantKG, users can see the variants that impact model decisions and use them for further downstream analysis. This will be demonstrated in Section 4.

### 3.11 Overall architecture

**Figure 7:**
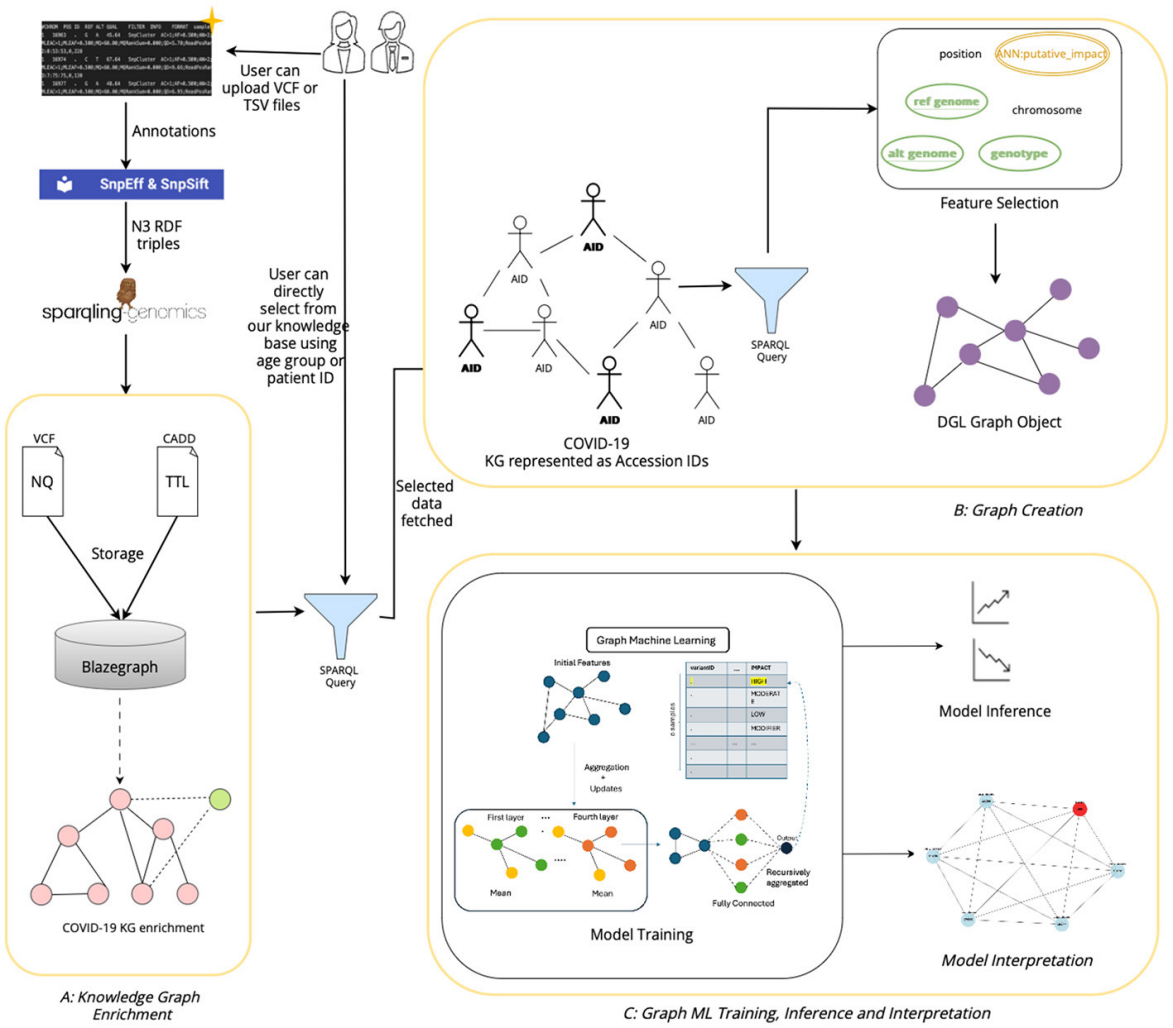
Architecture of VariantKG.

The architecture of our tool, shown in Figure 7 has been designed to facilitate the extraction, processing, and analysis of variant-level genomic data using GML. The workflow integrates custom data inputs, feature selection, storage in a graph database, graph creation, model training for inferencing genomic information and model interpretation. Given the rapidly expanding repository of genomic data globally, VariantKG offers a robust platform for researchers or users to extract relevant information and train GML models such as GraphSAGE, GCN, or Graph Transformer for inference purposes. Users have the flexibility to upload one or more VCF files, which may include associated CADD Scores for specific variants. Alternatively, users can select from our preexisting datasets that have been structured into the large-scale, comprehensive KG.

The first part of our tool, shown as *A: KG Enrichment* handles data upload and preprocessing, where users can upload one or more VCF with the associated CADD Scores or select from the preexisting database that contains genomic variants and annotations. If the user chooses to upload the files, the VCF and CADD Scores files are processed using the SnpEff tool to provide additional variant-level annotation and SPARQLing-Genomics for transforming the data into RDF using the *vcf2rdf* tool. The processed data is then stored along with the existing data on BlazeGraph, which is a scalable graph database. The data from the VCF files is stored as NQ RDF-quads, and the data from the CADD Scores files is stored as TTL (turtle) RDF-triples. This data is essentially incorporated into our existing knowledge base, enriching the KG with new information. The ontology defined for the KGs allows for this extensibility. Users also have the option to select the accession IDs corresponding to de-identified patients from our existing large-scale KG. This feature has been provided by utilizing an efficient, well-formed SPARQL query that fetches all the unique accession IDs that are available in the KG. This will be further discussed in Section 4.3.

The second part in the workflow of our tool, shown as *B: Graph Creation*, is the availability of feature selection and creation of the subgraph or graph suitable for model training. The user can select the features that are available in the NQ VCF files, which are passed through a well-formed SPARQL query that retrieves the selected features. The query results are temporarily stored in a columnar storage format optimized for analytical queries. The user can then assign a feature as the class label, facilitating supervised learning tasks such as node classification. Once the user selects one or more accession IDs from the KG, the user will then have an option to select the features based on the patient's variant-level information. The data along with the selected features and class labels is used to create a *DGLGraph* object that represents the graph as integers. This is essential as DGL necessitates that the input data be formatted into a framework-specific graph object. This will be further discussed in Section 4.4.

Once the graph has been constructed, users can then train the DGL GML models to gain a better understanding of the model performance through inferencing, shown as *C: Graph ML Training & Inference*. In our current implementation, we support GraphSAGE, GCN, and Graph Transformer models and intend to extend the support to other GML models in the future. The training process can be customized based on user-defined model hyperparameters. Existing data that have been prestructured as DGL graphs can also be loaded directly for model training. The user can observe the training process through the displayed charts and gain knowledge from the confusion matrices to assess the model performance during inference. This will be further discussed in Section 4.5. The users can also view the top features of trained model and explore the adjacency nodes of predicted node through model interpretability feature of the tool.

The UI was developed using Gradio (Abid et al., 2019), HTML5, and JavaScript, and the backend code was developed using Python 3.8.

## 4 Case study

In this section, we present a case study of how VariantKG can be used on a genomic dataset obtained from COVID-19 patients (Rao et al., 2021). We also report the performance evaluation of VariantKG by increasing the number of VCF files.

### 4.1 Experiment setup

The experiments were run on CloudLab (Ricci et al., 2014), an experimental testbed for cloud computing research. They were conducted on a bare metal machine with 2 Intel E5-2660 v2 (10 cores per CPU), 256 GB RAM, and two 1 TB disk drives. The machine ran Ubuntu 18.04.

### 4.2 Performance evaluation

We evaluated the performance of VariantKG using three GNN models: GraphSAGE, GCN, and Graph Transformer. As detailed in Table 5, we maintained consistent hyperparameters across all models, with 16 layers and a learning rate of 0.001. The models were trained for varying numbers of epochs (500, 1000, and 1500) to assess their performance over extended training periods. In general, GraphSAGE demonstrated significantly higher and more stable performance, maintaining an accuracy of 0.69 across 500, 1000, and 1500 epochs. This suggests that GraphSAGE may be more robust to extended training in the context of our VariantKG framework. It is important to note that all models utilized default features extracted from VCF files as input, with Variant Impact considered as the target label.

**Table 5:** A comparison of different GML models for VariantKG.

**Epochs**	**No. of layers**	**Model**	**Learning rate**	**Accuracy**
500	16	GraphSAGE	0.001	**0.69**
500	16	GCN	0.001	0.67
500	16	Graph Transformer	0.001	0.61
1000	16	GraphSAGE	0.001	**0.69**
1000	16	GCN	0.001	0.61
1000	16	Graph Transformer	0.001	0.60
1500	16	GraphSAGE	0.001	**0.69**
1500	16	GCN	0.001	**0.69**
1500	16	Graph Transformer	0.001	0.62

Best value shown in bold.

Table 6 presents a comparative analysis of VariantKG's performance with and without parallelization for VCF annotation and uploading to BlazeGraph. Essentially, we create a separate thread to process each VCF file independently. As a result, we can exploit many cores on the processor. The results demonstrate a significant improvement in processing time when parallelization was employed. For a small dataset of 10 VCF files, parallelization reduced the processing time from 546 seconds to 156.41 seconds, marking a 71.4% improvement. This efficiency gain becomes more pronounced as the dataset size increases. For instance, with 1000 VCF files, the processing time was reduced from 134,342 s (approximately 37.3 hours) without parallelization to 29,460.9 s (about 8.2 hours) with parallelization, representing a 78.1% reduction in processing time. The data consistently shows that parallelization offers substantial time savings across all dataset sizes, with the relative efficiency improvement ranging from 71.4% to 82.5%. This trend underscores the scalability of the parallelized approach, which is particularly beneficial for large-scale genomic data processing in VariantKG.

**Table 6:** VariantKG's performance with and without parallelization.

**No. VCF files**	**VCF annotation/uploading to BlazeGraph**	
	**Without parallelization**	**With parallelization**
10	546 s	156 s
50	2,750 s	775 s
100	6,450 s	1,646 s
500	47,101 s	8,230 s
1,000	134,342 s	29,460 s

Table 7 provides a comprehensive analysis of execution times for VariantKG across its different stages, showcasing its efficiency in handling increasing number of VCF files. The considered stages include VCF annotation and uploading to BlazeGraph and creating a DGL graph for ML. The creation of the DGL graph involves executing a SPARQL query followed by additional processing steps to create the necessary DataFrames. For instance, with 10 VCF files, the total execution time for VCF annotation and database storage was 156.41 s. As the number of VCF files increased, VariantKG achieved good performance. Even with 500 files, the VCF annotation and database storage stage completes in a reasonable 8,230 s, reflecting the robust design of VariantKG to scale with larger datasets. The DGL graph creation stage also showed good performance, with times increasing from 454 s for 10 files to 8,511 s for 500 files, indicating that VariantKG can handle large number of VCF files. For 1,000 VCF files, the SPARQL query did not finish even after 24 h when all the 30 features were selected. For such situations, we would recommend using a distributed RDF indexing and querying framework instead of BlazeGraph.

**Table 7:** Execution time for different stages in VariantKG.

**No. VCF files**	**VCF annotation + DB storage**	**Feature extraction (SPARQL-based)**	**DGL graph creation**
10	156 s	450 s	4 s
50	775 s	523 s	10 s
100	1,646 s	1,022 s	28 s
500	8,230 s	8,322 s	189 s
1,000	29,460 s	Failed to complete in 24 h	-

### 4.3 Scenario 1: KG enrichment

**Figure 8:**
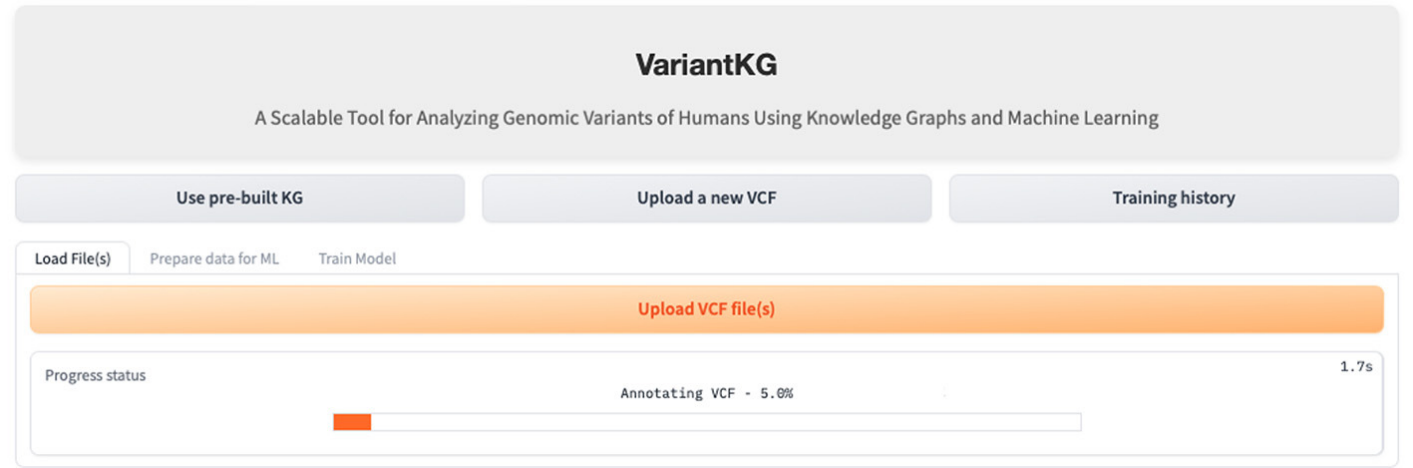
Screenshot showing how a user can upload new VCF files to enrich the KG.

The first part of the VariantKG tool focuses on KG enrichment. A user can upload one or more VCF files containing variant-level information and CADD Scores in a TSV format as shown in Figure 8 or the user can select patients from the existing KG. If the user uploads VCF files, the information in the files is first run through the SnpEFF tool. SnpEFF adds new annotated information to each variant in the VCF file and also updates the headers of the file to reflect the annotations. These annotations are functional information that is added to the ‘INFO' field using the ‘ANN' tag. This ANN field, in turn, consists of several bits of information, such as allele, annotation using Sequence and Ontology terms, putative impact, gene name, gene ID, feature type, feature ID, transcript biotype, exon or intron rank, cDNA position, protein position and several types of distances to the feature.

Once the files have been annotated, we then utilize the *vcf2rdf* tool provided by SPARQLing-Genomics to efficiently translate the information on each variant into several triples in an RDF-suitable N3 format. These N3 triples were converted to NQ format for several reasons. Converting to NQ format allows for the use of a named graph, which provides a robust way to group triples into distinct sets, considerably enhancing data organization and management. Another advantage of using named graphs is the support it provides to associate new metadata to specific data subsets easily. Additionally, named graphs also enable more precise and efficient SPARQL queries, improving data extraction quality and speed.

**Figure 9:**
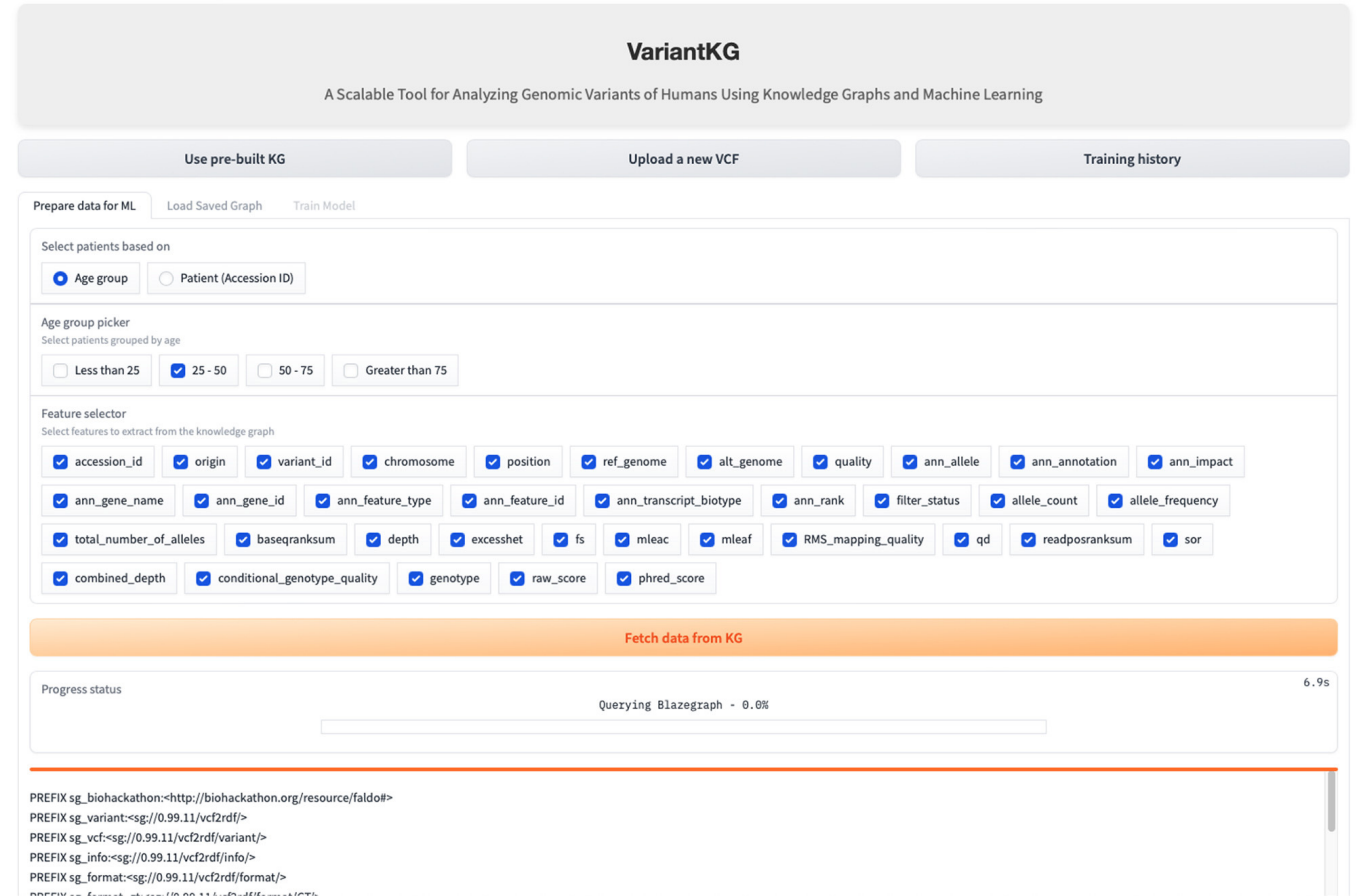
Screenshot showing how a user can select data from the KG based on age.

**Figure 10:**
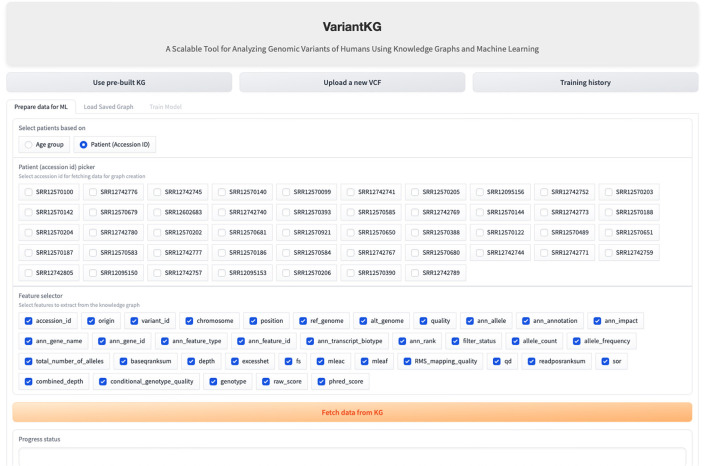
Screenshot showing how a user can select data from the KG based on existing patients.

The RDF data are stored in BlazeGraph, a high-performance graph database. To prepare the data for GML tasks, users can select patients using age groups as shown in Figure 9, or using the accession IDs, as shown in Figure 10, which are internally fetched using another SPARQL query that is, for efficiency purposes, only executed when the user wishes to prepare data for downstream GML tasks.

The user can then select the features from a list consisting of the original headers from the VCF files and the annotated features by SnpEff. Once the user hits the “Fetch from KG” button, another SPARQL query is then executed in the backend. If the user has selected from an age group, the SPARQL query consists of a filter that fetches all the accession IDs, which is passed as a list to the final query. If the user selects the accession IDs, it is passed as a list, similar to the previous query and the final query, shown below, fetches all the features selected for those patients or accession IDs.


 
      PREFIX sg_biohackathon: < http://biohackathon.org/resource/faldo#>
      PREFIX sg_variant: < sg://0.99.11/vcf2rdf/>
      PREFIX sg_vcf: < sg://0.99.11/vcf2rdf/variant/>
      PREFIX sg_info: < sg://0.99.11/vcf2rdf/info/>
      PREFIX sg_format: < sg://0.99.11/vcf2rdf/format/>
      PREFIX sg_format_gt: < sg://0.99.11/vcf2rdf/format/GT/>
      PREFIX ns1: < http://sg.org/>
  
      SELECT DISTINCT ?accession_id ?origin (COALESCE(?variant_id, ‘‘None'') AS ?variant_id) ?chromosome ?position ?ref_genome ?alt_genome ?quality ?ann ?ann_split_1 ?filter_status ?allele_count ?allele_frequency ?total_number_of_alleles ?baseqranksum ?depth ?excesshet ?fs ?mleac ?mleaf ?RMS_mapping_quality ?qd ?readposranksum ?sor ?combined_depth ?conditional_genotype_quality ?genotype ?raw_score ?phred_score
      WHERE {
        GRAPH ?accession_id {
          OPTIONAL { ?origin sg_variant:variantId ?variant_id . }
          BIND (COALESCE(?variant_id, ‘‘None'') AS ?variant_id)
          ?origin sg_biohackathon:reference ?chromosome .
          ?origin sg_biohackathon:position ?position .
          ?origin sg_vcf:REF ?ref_genome .
          ?origin sg_vcf:ALT ?alt_genome .
          ?origin sg_vcf:QUAL ?quality .
          ?origin sg_info:ANN ?ann .
          BIND (IF(STRLEN(?ann) - STRLEN(REPLACE(?ann, ‘‘,'', ‘‘'')) = 0, ?ann, STRBEFORE(?ann, ‘‘,'')) AS ?ann_split_1)
          OPTIONAL { ?origin sg_info:FILTER_STATUS ?filter_status . }
          OPTIONAL { ?origin sg_info:ALLELE_COUNT ?allele_count . }
          OPTIONAL { ?origin sg_info:ALLELE_FREQUENCY ?allele_frequency . }
          OPTIONAL { ?origin sg_info:TOTAL_NUMBER_OF_ALLELES ?total_number_of_alleles . }
          OPTIONAL { ?origin sg_info:BASEQRANKSUM ?baseqranksum . }
          OPTIONAL { ?origin sg_info:DEPTH ?depth . }
          OPTIONAL { ?origin sg_info:EXCESSHET ?excesshet . }
          OPTIONAL { ?origin sg_info:FS ?fs . }
          OPTIONAL { ?origin sg_info:MLEAC ?mleac . }
          OPTIONAL { ?origin sg_info:MLEAF ?mleaf . }
          OPTIONAL { ?origin sg_info:RMS_MAPPING_QUALITY ?RMS_mapping_quality . }
          OPTIONAL { ?origin sg_info:QD ?qd . }
          OPTIONAL { ?origin sg_info:READPOSRANKSUM ?readposranksum . }
          OPTIONAL { ?origin sg_info:SOR ?sor . }
          OPTIONAL { ?origin sg_info:COMBINED_DEPTH ?combined_depth . }
          OPTIONAL { ?origin sg_info:CONDITIONAL_GENOTYPE_QUALITY ?conditional_genotype_quality . }
          OPTIONAL { ?origin sg_info:GENOTYPE ?genotype . }
          OPTIONAL { ?origin sg_info:RAW_SCORE ?raw_score . }
          OPTIONAL { ?origin sg_info:PHRED_SCORE ?phred_score . }
        }
        ?origin sg_biohackathon:position ?position .
        OPTIONAL { ?variant  < http://sg.org/has_pos> ?position . }
        ?origin sg_vcf:REF ?ref_genome .
        OPTIONAL { ?variant  < http://sg.org/has_ref_genome> ?ref_genome . }
        ?origin sg_vcf:ALT ?alt_genome .
        OPTIONAL { ?variant  < http://sg.org/has_alt_genome> ?alt_genome . }
        OPTIONAL { ?variant  < http://sg.org/has_cadd_scores> ?cadd_scores . }
        OPTIONAL { ?cadd_scores  < http://sg.org/has_raw_score> ?raw_score . }
        OPTIONAL { ?cadd_scores  < http://sg.org/has_phred> ?phred_score . }
        FILTER (?accession_id IN (accession_id_list))
      } ORDER BY ?variant_id
 


### 4.4 Scenario 2: graph creation

**Figure 11:**
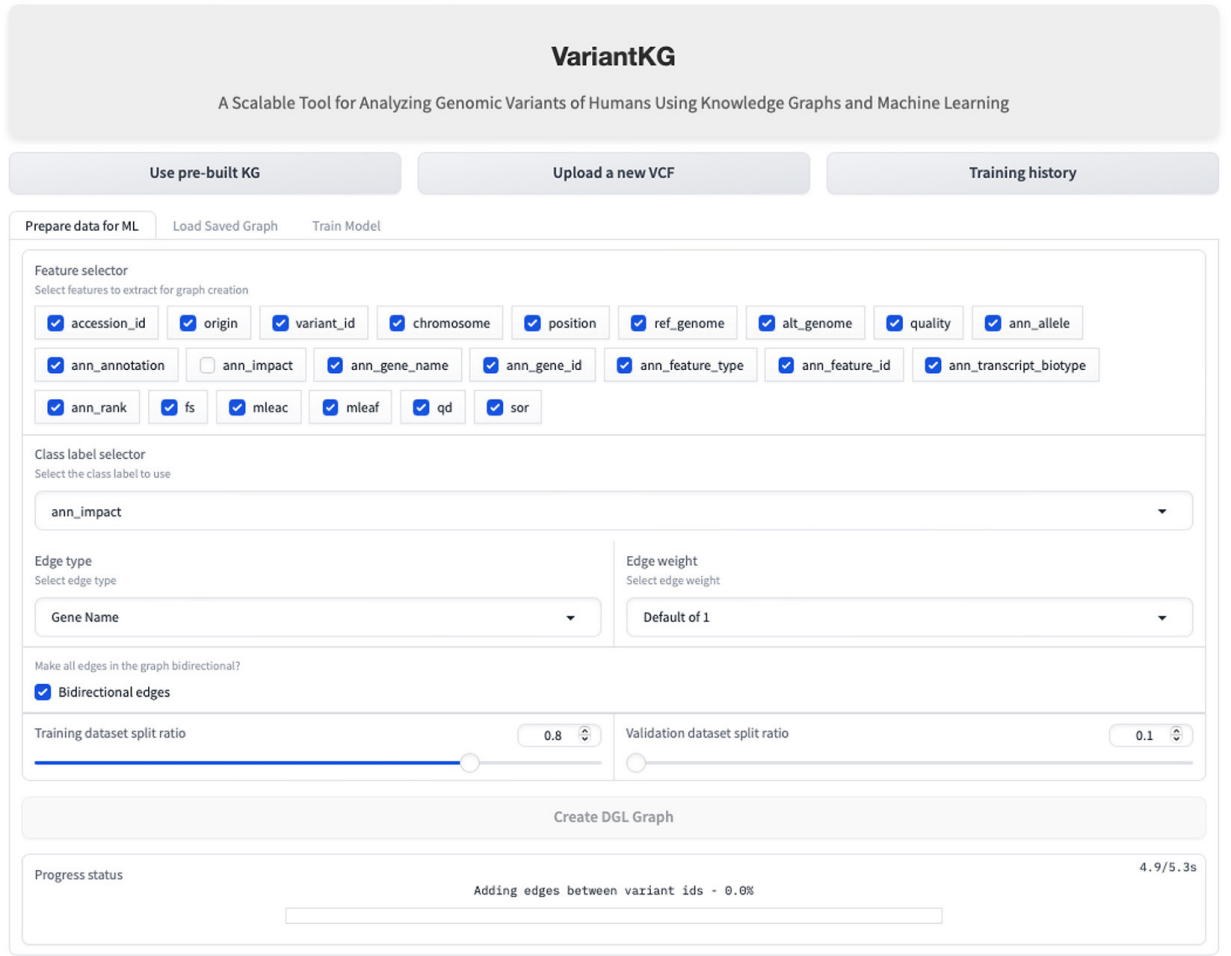
Screenshot showing how a user can create the DGL graph for the GML classification task.

Once the data has been fetched in the backend, the user is redirected to ‘Graph Creation' where the user can select node features in the graph and select the class label from a subset of the meaningful features to be classified as a part of the classification task. The user is then given complete control of the graph construction where the user can select the edge type, which can be Gene Name (default) or fully connected. It is important to note that the graphs are homogeneous in nature, containing only one edge type. The weight of the edges can be 1 (default), the number of incoming edges to a node, or a user-defined value. The user can then select if the edges should be bidirectional and the train:validation splits. The default is 80:10 with the remaining 10% calculated in the backend to minimize user clicks. Using this information, the graph is created for the node classification task. This is shown in Figure 11.

### 4.5 Scenario 3: GML training and inference

**Figure 12:**
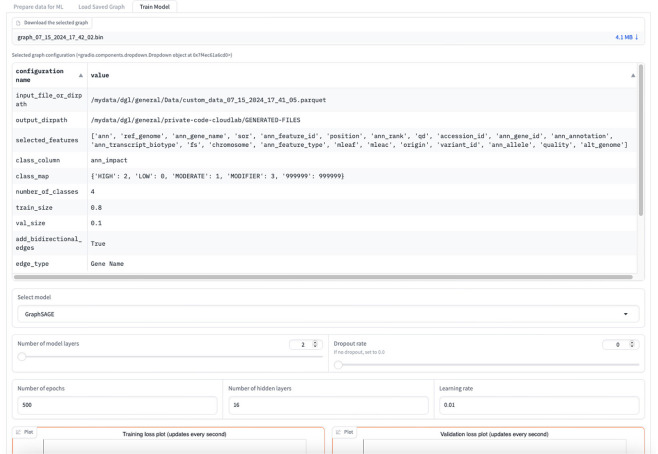
Screenshot during GML training.

The graph for the node classification task needs to be in a DGL-specific input format. The data is thus converted to a “DGLGraph” object that consists of only integers that are mapped to the actual string values. The user can view the summary of the graph once created. This summary consists of all the selected features, the class label, the number of classes, graph properties, and the number of nodes and edges. The user is given a choice to download the DGL graph or continue with the GML task. For the GML task, the user can select between GraphSAGE, GCN, and Graph Transformer models for the classification task and set the hyperparameters to train the model as shown in Figure 12. This includes the number of layers other than the input and output layers that constitute the primary architecture of the model, the number of hidden layers, the dropout rate, the learning rate, and the number of epochs for training.

**Figure 13:**
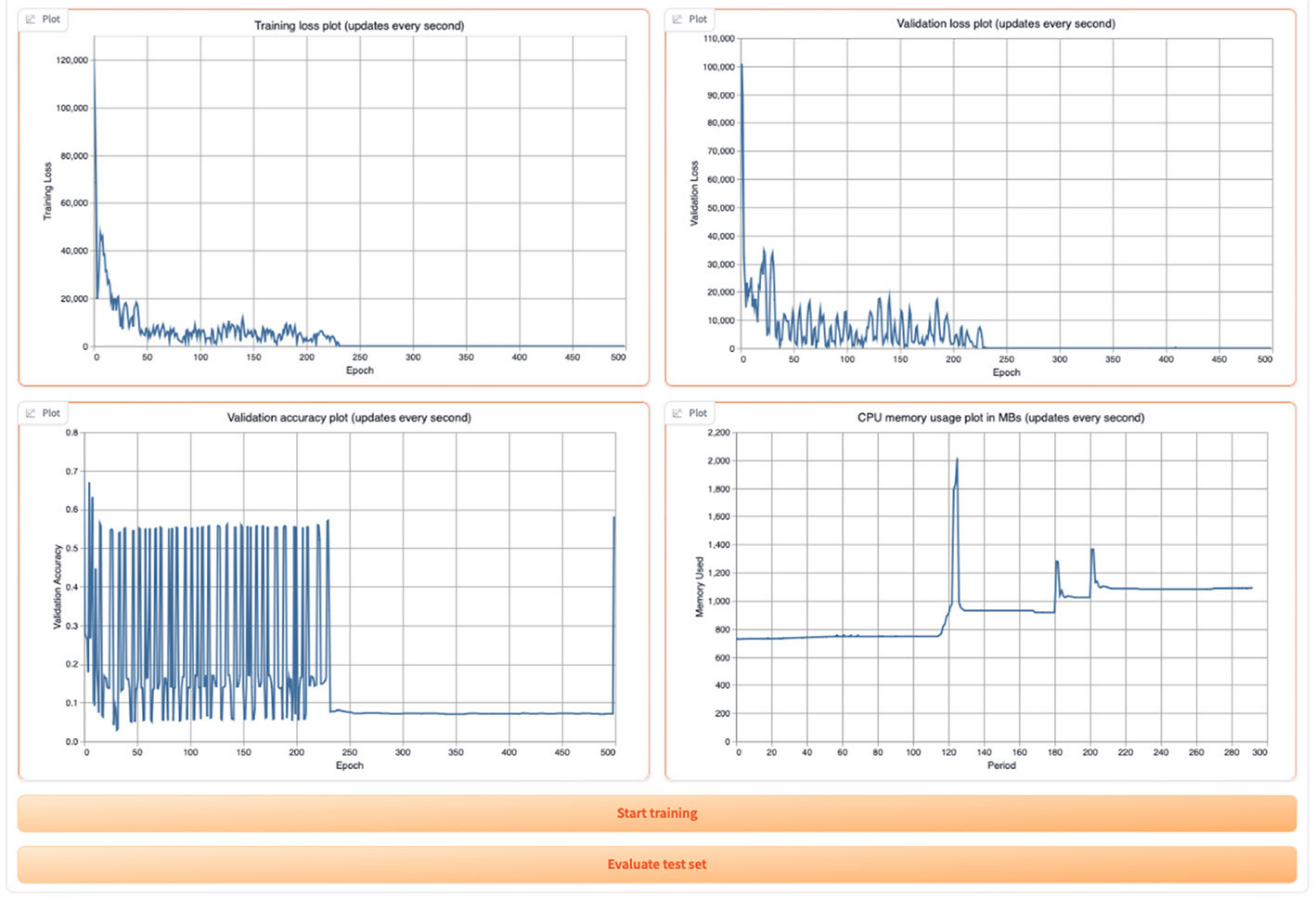
Display of GML training and validation loss, validation accuracy, and CPU usage in the tool.

Once the training begins, the user can view the training and validation loss plots, the validation accuracy plot, and the CPU memory usage as shown in Figure 13.

**Figure 14:**
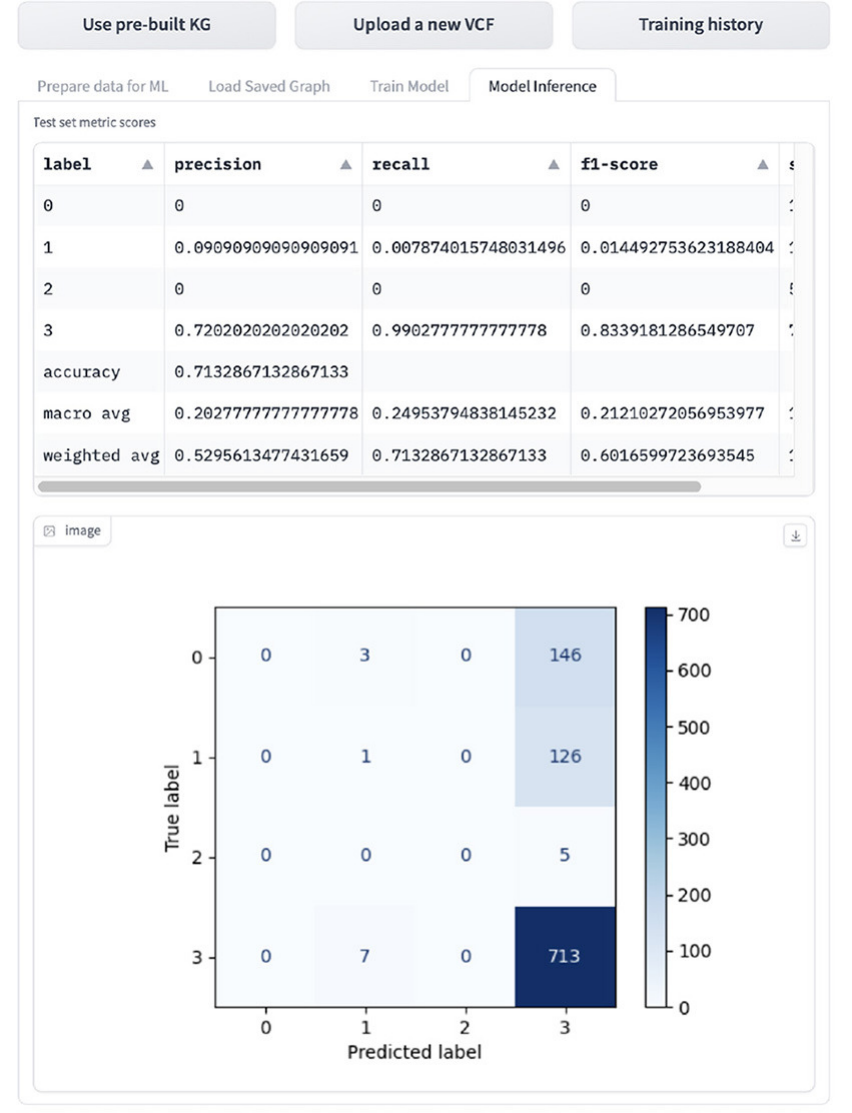
Display of GML inference results in the tool.

If the user wants to infer the GML task, they can navigated to the “Inference” tab shown in Figure 14, which displays the evaluation metrics and confusion matrix. The model is evaluated on accuracy, macro- and weighted-precision, recall and F1 score, and support, which is the number of samples for the given class.

### 4.6 Scenario 4: model interpretation and important features

For interpretability, VariantKG uses the graph visualization and model interpretation capabilities of GNNExplainer (Ying et al., 2019) in DGL. VariantKG offers a *Model Interpretation* feature that allows users to customize their analysis by specifying three key parameters:

- Degree/hops per node: this determines the number of connected nodes.
- Number of subgraphs to display: given the potentially large number of possible subgraphs, users can select how many they wish to view.
- Node ID: this option enables users to focus on and explore the features of a specific target node.

**Figure 15:**
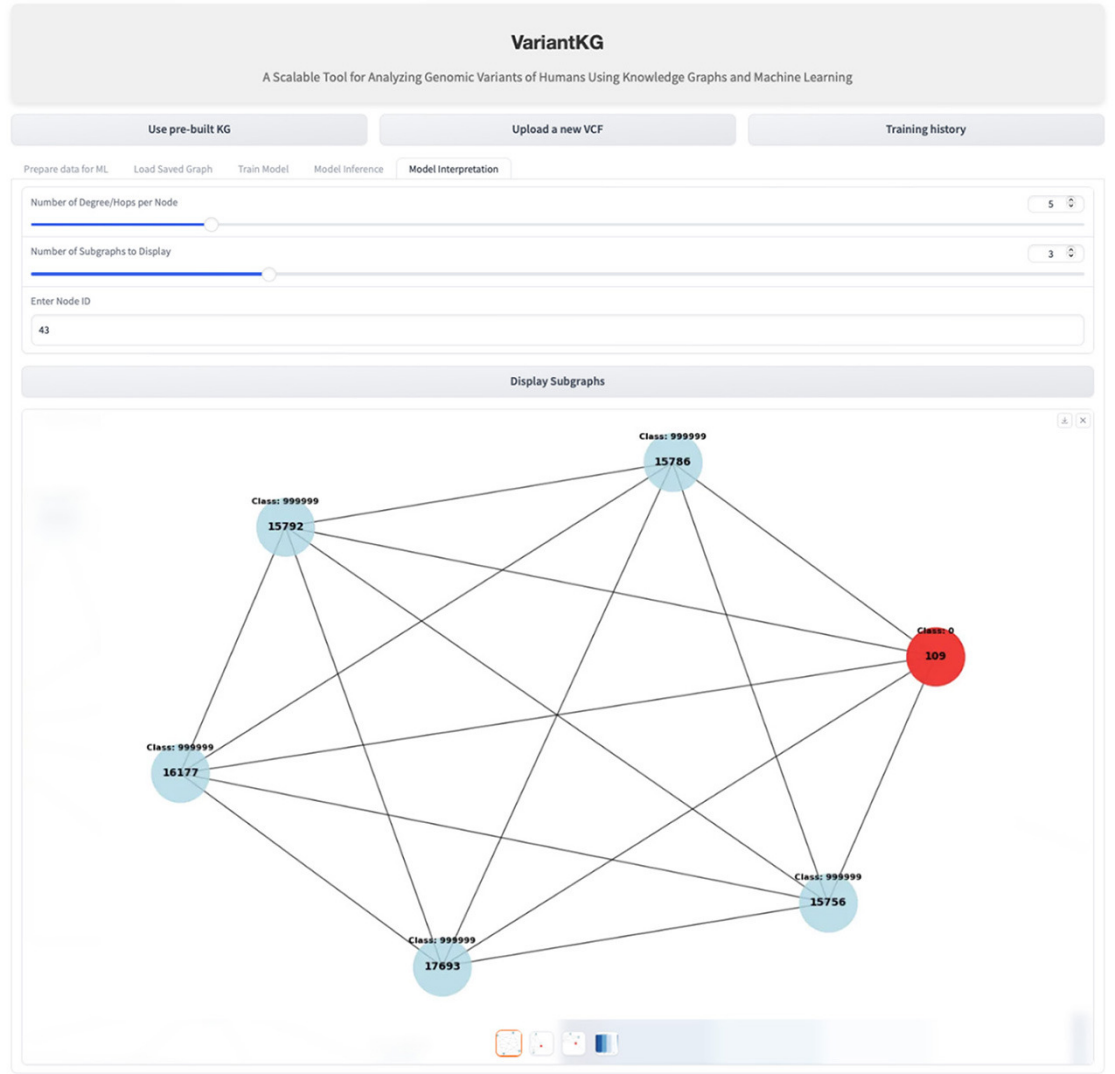
Screenshot showing GML model interpretation.

These parameters provide flexibility in visualizing the model's interpretability, allowing for a tailored examination of the graph structure and node relationships relevant to the prediction task. Figure 15 shows an example of interpretability for a node shown in red.

## 5 Conclusion

In this article, we demonstrated that representing genomic data as knowledge graphs enables integrating diverse information to understand complex human diseases better. We effectively organized and integrated genomic variant data from COVID-19 patients. Using our tool, VariantKG, which consists of several important components. Firstly, through a detailed data collection pipeline, we can extract large amount of genetic information on de-identified COVID-19 patients, followed by variant annotation using the SnpEff tool and then conversion into RDF format with the SPARQLing Genomics tool. We developed an ontology to standardize and collate the gathered information, which was used to construct a comprehensive KG. This large KG, further enriched with patient metadata such as age, sex, and disease stage, enables efficient querying and indexing through a scalable database, such as BlazeGraph. Additionally, we showcased the utility of this KG for node classification tasks by leveraging DGL within VariantKG's framework. Through a series of user scenarios, we successfully demonstrated how the tool allows for data extraction, training, and testing of KG subsets, thereby offering an easy-to-use and efficient tool for researchers. As a part of future work, we aim to further enlarge our KG by consuming more data and then performing graph machine learning tasks in a distributed setting.

## Data Availability

The datasets presented in this study can be found in online repositories. The names of the repository/repositories and accession number(s) can be found in the article/supplementary material. VariantKG software is available at https://github.com/MU-Data-Science/GAF.

## Footnotes

^1^ https://www.ncbi.nlm.nih.gov/sra

^2^ https://hadoop.apache.org/

^3^ https://spark.apache.org/
